# Cytokines regulate complement receptor immunoglobulin expression and phagocytosis of *Candida albicans* in human macrophages: A control point in anti-microbial immunity

**DOI:** 10.1038/s41598-017-04325-0

**Published:** 2017-06-22

**Authors:** Usma Munawara, Annabelle G. Small, Alex Quach, Nick N. Gorgani, Catherine A. Abbott, Antonio Ferrante

**Affiliations:** 1 School of Biological Sciences, Flinders University, Bedford Park, South Australia Australia; 2 Department of Immunopathology, SA Pathology at Women’s and Children’s Hospital, The Robinson Research Institute and Discipline of Paediatrics and Department of Cellular and Molecular Biology, University of Adelaide, Adelaide, Australia; 3 Children’s Medical Research Institute, Westmead, New South Wales Australia; 4 School of Pharmacy and Medical Sciences, University of South Australia, Adelaide, South Australia Australia; 5 OzStar Therapeutics Pty Ltd., Castle Hill, New South Wales Australia

## Abstract

Complement Receptor Immunoglobulin (CRIg), selectively expressed by macrophages, plays an important role in innate immunity by promoting phagocytosis of bacteria. Thus modulation of CRIg on macrophages by cytokines can be an important mechanism by which cytokines regulate anti-microbial immunity. The effects of the cytokines, tumor necrosis factor, transforming growth factor-β1, interferon-γ, interleukin (IL)-4, IL-13, IL-10, IL-1β, IL-6, lymphotoxin-α, macrophage-colony stimulating factor (M-CSF) and GM-CSF on CRIg expression were examined in human macrophages. We demonstrated that cytokines regulated the CRIg expression on macrophages during their development from monocytes in culture at the transcriptional level using qPCR and protein by Western blotting. Both CRIg spliced forms (Long and Short), were similarly regulated by cytokines. Direct addition of cytokines to matured CRIg+ macrophages also changed CRIg mRNA expression, suggesting that cytokines control macrophage function via CRIg, at two checkpoints. Interestingly the classical complement receptors, CR3 and CR4 were differentially regulated by cytokines. The changes in CRIg but not CR3/CR4 mRNA expression correlated with ability to phagocytose *Candida albicans* by macrophages. These findings suggest that CRIg is likely to be a control point in infection and immunity through which cytokines can mediate their effects, and is differentially regulated from CR3 and CR4 by cytokines.

## Introduction

Members of complement, Toll-like and scavenger receptors as well as C-type lectins are amongst the groups of receptors that initially recognize opsonised-pathogen or pathogen-associated molecular patterns. In the last decade, the B7 family-related protein V-set and Ig domain-containing 4 (VSIG4) (Z39Ig)^1–3^, was found to be an important complement (CRIg) receptor^4^. This receptor differs structurally and functionally from the classical complement receptors, CR3 and CR4. CRIg is expressed selectively by macrophages and is involved in the rapid phagocytosis of complement (C3b/iC3b)-opsonised pathogens^5^. The presence of CRIg on Kupffer cell surfaces results in the rapid uptake of circulating *Listeria monocytogenes* and *Staphylococcus aureus*, thereby limiting bacterial dissemination and pathogenesis^4^. CRIg^−/−^ mice infected with these bacteria exhibited exaggerated levels of inflammatory cytokines, and died earlier than wild type mice. More recently the uniqueness of this receptor in the clearance of bacteria by Kupffer cells was evident by showing that it promoted bacterial clearance by a dual track system in a complement dependent manner^6^ and clearance of gram-positive bacteria via non-complement ligands^6, 7^. While the function of CRIg in immunity to infection appears well established, there is little known about the modulation of expression of CRIg by inflammatory mediators generated during infection and inflammation.

It has been previously reported that monocytes in culture begin to express CRIg as they differentiate into macrophages^4, 8^. The development of CRIg^+^ macrophages was found to be up- (IL-10) or down (IFN-γ, IL-4 and TGF-β1) regulated by cytokines based on CRIg mRNA levels. The purpose of our investigation was to extend the work to include other important cytokines generated during infection and inflammation: IL-13, IL-1β, IL-6, lymphotoxin-α, M-CSF and GM-CSF, examining whether this relates to changes in CRIg protein expression level to enable us to evaluate the effects on the two spliced forms of CRIg, as well as assessing effects on mature macrophages. Since CRIg is likely to co-exist with CR3 and CR4 on these macrophages, comparisons were made with the expression of these receptors. Finally the cytokine-induced modulation of expression of these complement receptors was examined in the context of their anti-microbial action against complement opsonised *Candida albicans*.

## Results

We have previously shown that cultured human monocytes displayed maximal increase in CRIg mRNA expression on day 3 of culture and protein on day 5–7^8^. We confirmed these results in preliminary experiments (data not presented). Thus experiments were designed around these time points for examining the effects of cytokines on macrophage CRIg expression. Cytokines known to regulate macrophage function and which are produced in inflammatory sites were evaluated for their effects on CRIg expression.

### Effect of cytokines on the development of CRIg^+^ macrophages

**Figure 1:**
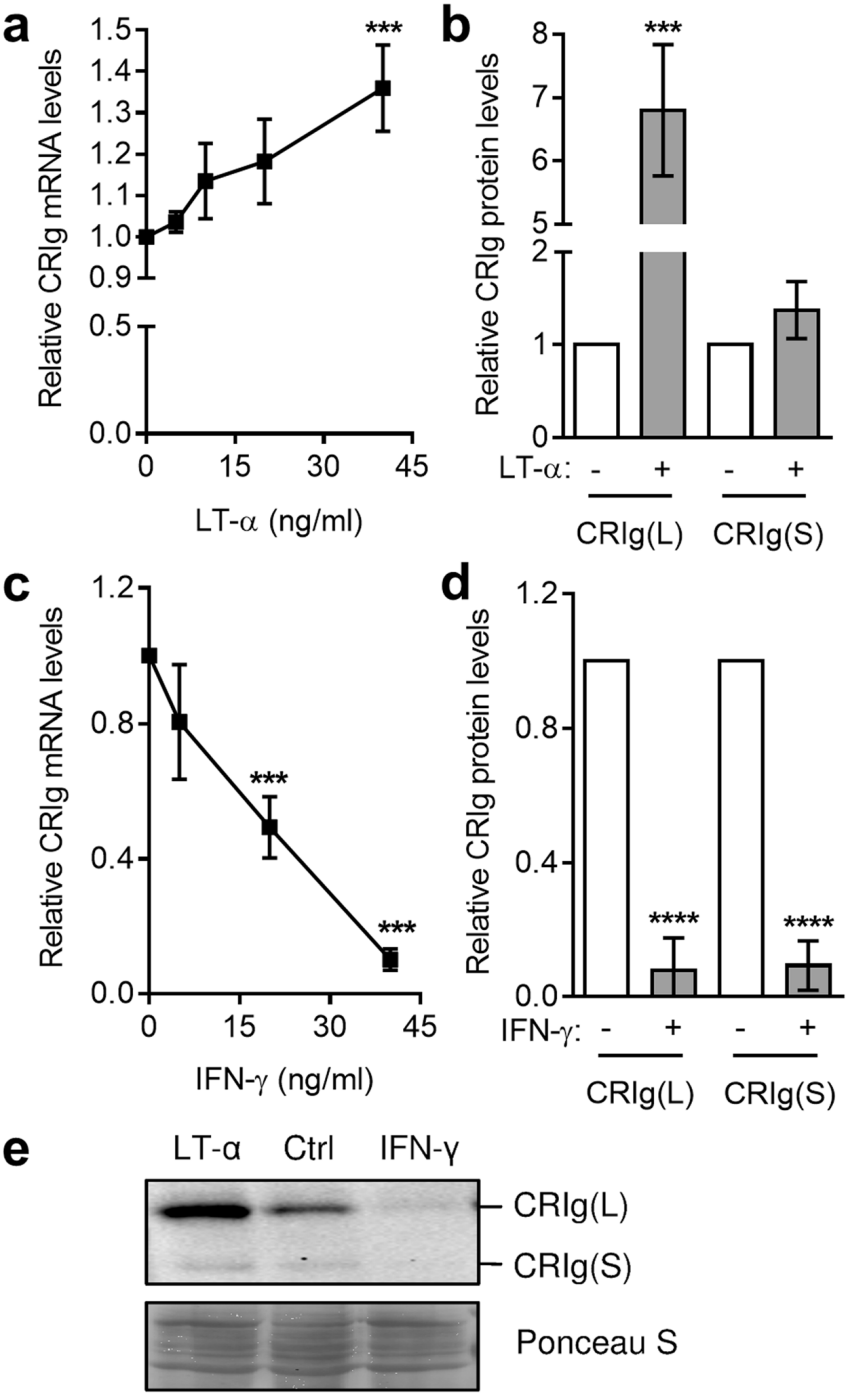
The development of CRIg^+^ macrophages is differentially modulated by LT-α and IFN-γ. Monocytes were cultured in the presence of 0, 5, 10, 20, and 40 ng/ml LT-α (**a**) or 0, 10, 20, and 40 ng/ml IFN-γ (**c**) for 3 days and then CRIg mRNA expression measured. Data are expressed as fold-change over GAPDH-normalised CRIg mRNA in the absence of cytokine set as 1. For CRIg protein expression monocytes were treated with 40 ng/ml LT-α (**b**,**e**) or IFN-γ (**d**,**e**) for 7 days and then the CRIg protein levels measured. Note both the Long and Short forms of CRIg are expressed. (**e**) A representative Western blot of total protein lysates is shown with Ponceau S staining showing consistency of protein load. Data are expressed as fold-difference in CRIg band intensity as determined by densitometry with CRIg expression in the absence of cytokine set as 1. Data are presented as means ± SD of three experiments each conducted with cells from three different individuals. ***p < 0.001, ****p < 0.0001.

**Figure 2:**
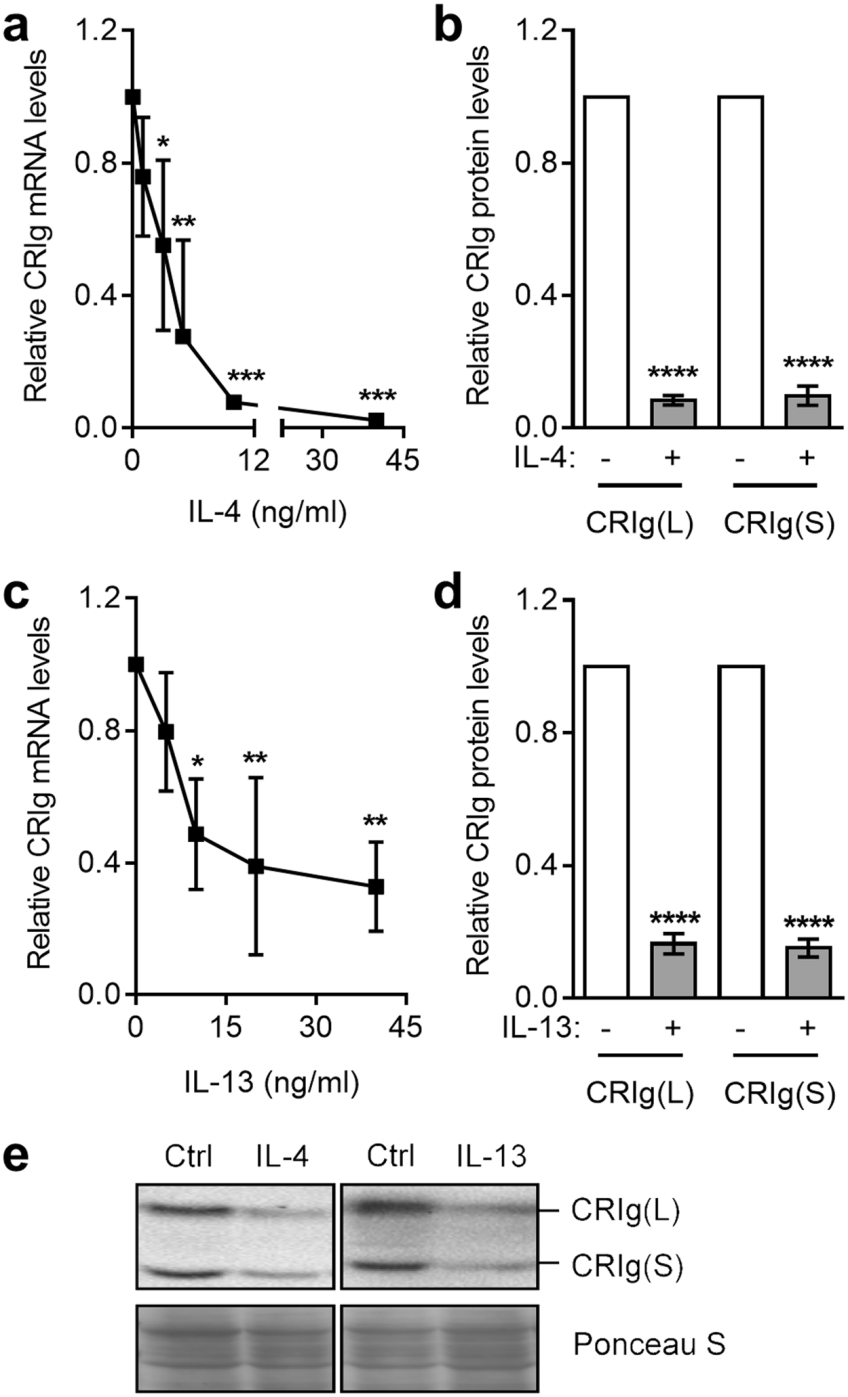
IL-4 and IL-13 down-regulate the development of CRIg^+^. Monocytes were cultured in the presence of 0, 1, 3, 5, 10, and 40 ng/ml IL-4 (**a**) or 0, 5, 10, 20, and 40 ng/ml IL-13 (**c**) and CRIg mRNA expression was measured by qPCR. For CRIg protein expression the monocytes were treated with 40 ng/ml (**b**) IL-4 or IL-13 (**d**). (**e**) Representative Western blots of CRIg levels (IL-4 and IL-13 treatments were analysed on separate blots). Data are presented as means ± SD of three experiments, each conducted with cells from different individuals. *p < 0.05, **p < 0.01, ***p < 0.001, ****p < 0.0001.

Monocytes were cultured in the presence of either the Th1 cytokines, LT-α and IFN-γ or the Th2 cytokines IL-4 and IL-13 and then examined for levels of CRIg mRNA after 3 days by qPCR and protein at day 7 by Western blot analysis using anti-CRIg antibody. In the presence of LT-α there was an increase in CRIg mRNA and marked increase in CRIg protein (Fig. 1a and b). In contrast, IFN-γ caused a substantial decrease in CRIg mRNA and protein expression (Fig. 1c and d). These effects were seen over a concentration range of 5–40 ng/ml for LT-α and 10–40 ng/ml for IFN-γ. The Th2 cytokines, IL-4 and IL-13 both markedly inhibited the expression of CRIg at the mRNA and protein levels (Fig. 2). The effects occurred in a concentration range of 1–40 ng/ml for IL-4 and 5–40 ng/ml for IL-13. Western blot analysis enabled us to distinguish between the two different forms of CRIg, the long (L) and short (S) forms (Fig. S1). The data in Figs 1(b,d,e) and 2(b,d,e) showed that the two forms were similarly regulated by the cytokines. It is also evident that CRIg(L) is the more prominent form in these macrophages, even after treatment with cytokines.

**Figure 3:**
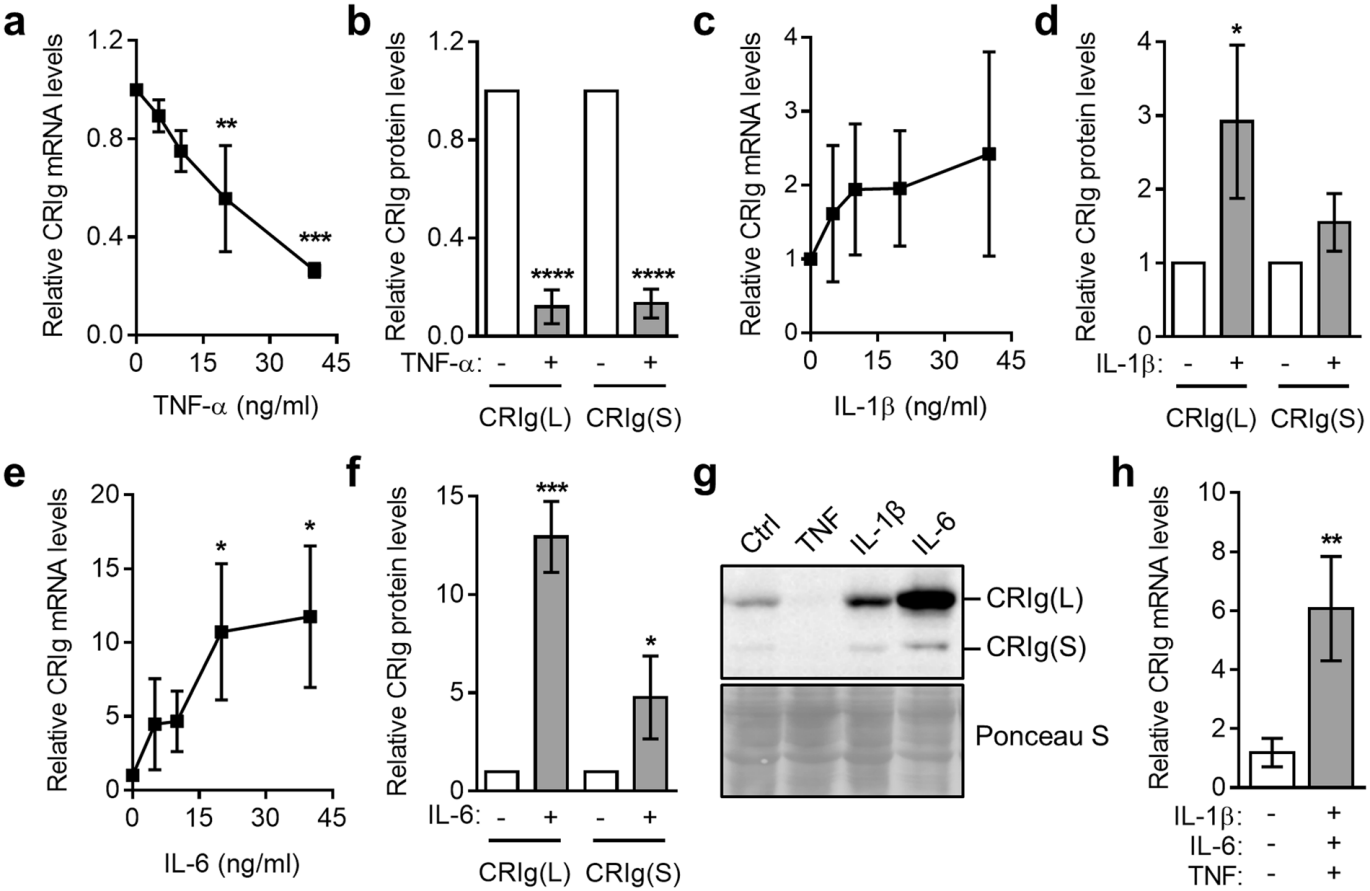
The pyrogenic cytokines, TNF, IL-1β and IL-6 differentially regulate CRIg^+^ macrophage development. Monocytes were cultured in the presence of 0, 5, 10, 20, 40 ng/ml TNF (**a**), IL-1β (**c**) or IL-6 (**e**) and CRIg mRNA expression measured by qPCR. For CRIg protein expression monocytes were treated with 40 ng/ml TNF (**b**), IL-1β (**d**) or IL-6 (**f**). (**e**) A representative Western blot of CRIg levels and total protein in lysates is shown. (**h**) shows the effect of combined addition of 40 ng/ml of TNF, IL-1β and IL-6 to the development of CRIg^+^ MDM. Data are presented as means ± SD of three experiments, each conducted with cells from different individuals. *p < 0.05, **p < 0.01, ***p < 0.001, ****p < 0.0001.

TNF, IL-1β and IL-6 are cytokines referred to as pyrogenic and pro-inflammatory cytokines which predominate during infection and inflammation, associated with chronic inflammatory diseases. Because of the importance of CRIg in phagocytosis and regulation of inflammation, their effects on CRIg expression in cultured macrophages were examined. Treatment of monocytes with TNF caused a marked reduction of CRIg mRNA and protein in the maturing macrophages (Fig. 3a and b). This reduced expression occurred in a concentration dependent manner. In relation to CRIg protein expression, TNF caused approximately 80% reduction. IL-1β, and in particular IL-6 increased CRIg expression in macrophages (Fig. 3c–f). CRIg protein expression analysed by Western blotting demonstrated that expression of both forms, L and S, were altered in a similar manner in cells cultured in the presence of these cytokines (Fig. 3b,d,f,g). In order to gain more physiological meaningful information in regards to cytokine profiles and CRIg expression on macrophages, mixtures of cytokines that are up-regulated in bacterial infections and chronic inflammatory conditions such as rheumatoid arthritis were examined, namely IL-1β, IL-6 and TNF. When monocytes were cultured in the presence of this mixture of cytokines, there was a resultant increase in expression of CRIg during their development i.e. the down regulation induced by TNF was overcome by having IL-1β and IL-6 present (Fig. 3h).

**Figure 4:**
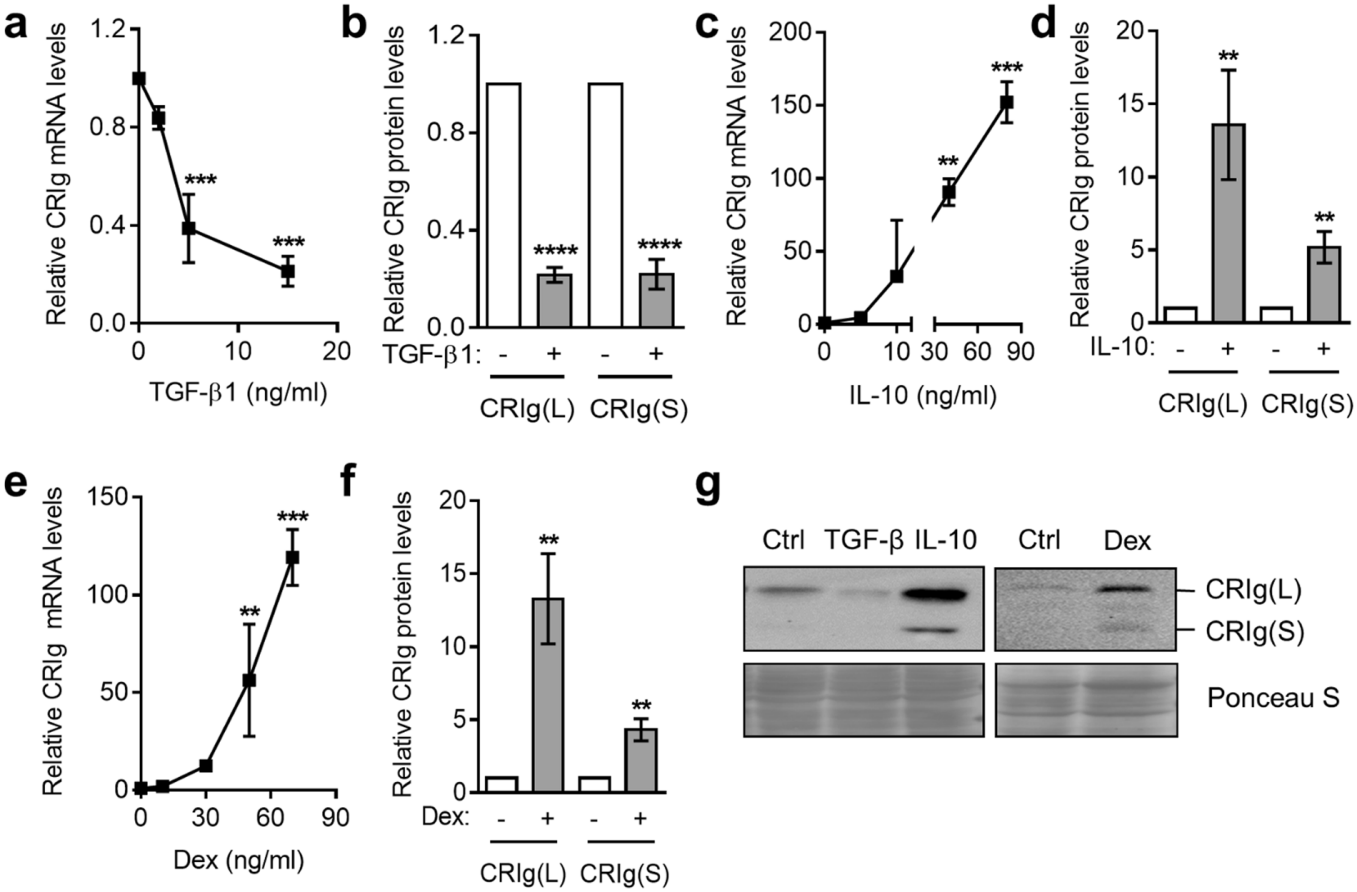
The effect of regulatory cytokines TGF-β1 and IL-10 on CRIg^+^ macrophage development. Monocytes were cultured in the presence of 0, 2, 5, 15 ng/ml TGF-β1 (**a**) or 0, 5, 10, 40, 80 ng/ml IL-10 (**c**) and CRIg mRNA expression was measured by qPCR. For CRIg protein expression monocytes were treated with 15 ng/ml TGF-β1 (**b**) or 40 ng/ml IL-10 (**d**). (**e**) Monocytes were cultured in the presence of dexamethasone and the CRIg mRNA determined. (**f**) For protein expression monocytes were treated with 30 ng/ml dexamethasone. (**g**) Representative blots are shown (dexamethasone treatment was analysed on a separate blot from TGF-β1 and IL-10). Data are presented as means ± SD of three experiments, each conducted with cells from different individuals. **p < 0.01, ***p < 0.001, ****p < 0.0001.

TGF-β1 and IL-10 share a number of properties and have been shown to regulate and depress inflammation. Their effects on macrophage function have been reported. We have now examined their effects on CRIg mRNA and protein expression. Culturing monocytes with TGF-β1 led to a concentration (2–15 ng/ml) related decrease in CRIg mRNA expression with almost complete suppression of CRIg protein expression (Fig. 4a and b). In contrast IL-10 caused a marked increase in CRIg expression in macrophages (Fig. 4c and d). When we compared this with the effects of dexamethasone it was evident that IL-10 was as effective as dexamethasone in increasing CRIg expression (Fig. 4e and f). This was seen at both the mRNA and protein level. Although the effects of dexamethasone on total CRIg cellular protein was not previously studied^8^ it is evident from the Western blot analysis that the steroid increased the cellular expression of both CRIg(L) and CRIg(S) forms (Fig. 4f). Both forms of CRIg were similarly regulated by TGF-β1 and IL-10 (Fig. 4b and d).

**Figure 5:**
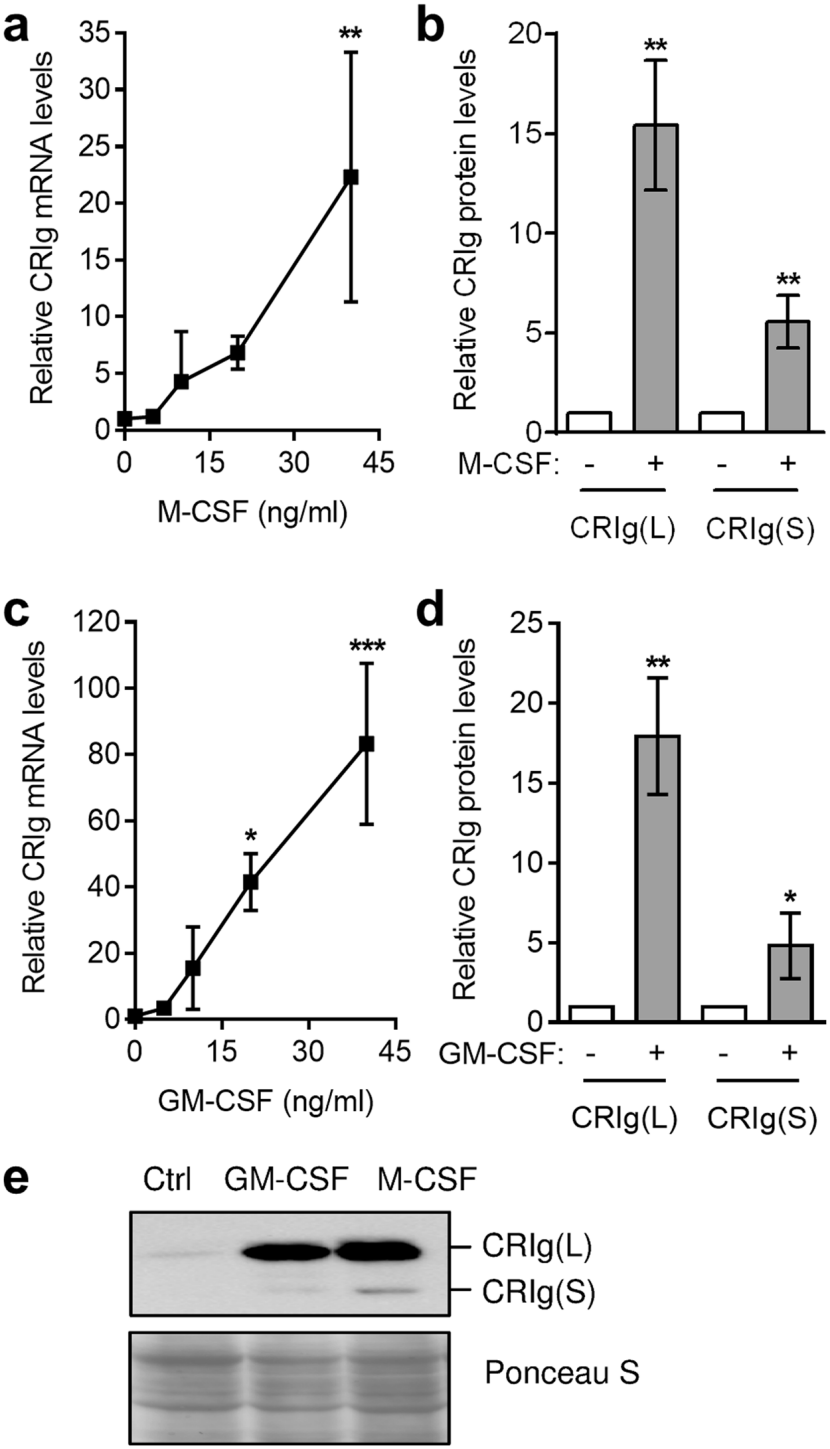
M-CSF and GM-CSF promote the development of CRIg^+^ macrophages. Monocytes were cultured in the presence of 0, 5, 10, 20, 40 ng/ml M-CSF (**a**) or GM-CSF (**c**). Then CRIg mRNA expression measured. For CRIg protein expression monocytes were treated with 40 ng/ml M-CSF (**b**) or GM-CSF (**d**). (**e**) A representative Western blot. Data are presented as means ± SD of three experiments, each conducted with cells from different individuals, *p < 0.05, **p < 0.01, ***p < 0.001.

We extended our studies to another set of cytokines which are involved in controlling macrophage function, M-CSF and GM-CSF. When monocytes were cultured in the presence of these cytokines, both caused an increase in CRIg mRNA and protein expression in the macrophage population (Fig. 5). Both of these cytokines caused a marked increase in expression, comparable to that induced by IL-10. The expression of both CRIg L and S forms was increased by M-CSF and GM-CSF (Fig. 5b and d).

### The effect of cytokines on CRIg expression on mature macrophages (MDM)

In the previous section we have presented data which resulted from examining the effects of cytokines on the development of CRIg^+^ macrophages, monocyte-derived macrophages (MDM). While this forms one stage of understanding of how mediators control CRIg expression in macrophages in particular during inflammation and monocytes infiltration into tissues, it does not reveal whether mature macrophages present already expressing CRIg can be modulated by cytokines. Thus a second stage for regulating inflammation is for cytokines to act on already mature macrophages, such as MDM.

**Figure 6:**
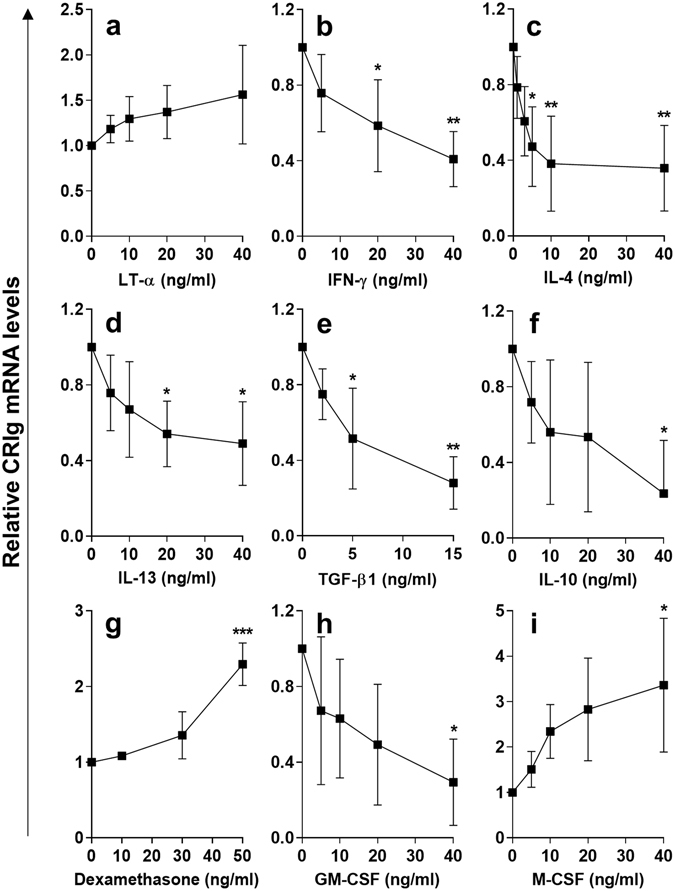
Effects of cytokines on CRIg expression in matured macrophages (MDM). In these studies MDM were prepared by culturing human monocytes for 3 days. MDM from 3 day cultures were treated with LT-α (**a**) (0, 5, 10, 20 and 40 ng/ml) or IFN-γ (**b**) (0, 5, 20 and 40 ng/ml) or IL-4 (**c**) (0, 1, 3, 5, 10 and 40 ng/ml) or IL-13 (**d**) (0, 5, 10, 20 and 40 ng/ml), TGF-β1 (**e**) (0, 2, 5 and 15 ng/ml) or (**f**) IL-10 (0, 5, 10, 20 and 40 ng/ml) or M-CSF/GM-CSF (**g**,**h**) (0, 5, 10, 20 and 40 ng/ml) or dexamethasone (**i**) (0, 10, 30 and 50 ng/ml) for 24 h and then CRIg mRNA levels relative to GAPDH mRNA were assessed by qPCR. Data are expressed as fold-change over GAPDH-normalised CRIg mRNA in the absence of cytokine set as 1. Data are presented as means ± SD of three experiments, each conducted with cells from different individuals, *p < 0.05, **p < 0.01, ***p < 0.001.

MDM expressing CRIg were generated from monocytes in culture in the absence of cytokines. The MDM were then examined to see what effects cytokines had on expression of CRIg mRNA expression. The macrophages were treated with the cytokines for 24 h and then examined for levels of CRIg mRNA. Treatment with 5–40 ng/ml of LT-α caused an increase in CRIg mRNA (Fig. 6a). In comparison, another Th1 cytokine IFN-γ caused a marked decrease in CRIg mRNA expression over a concentration range of 5–40 ng/ml reaching a decrease of approximately 60% at 40 ng/ml (Fig. 6b). TNF is also considered a Th1 lymphocyte cytokine. Under these same conditions TNF caused a substantial decrease in CRIg mRNA expression, compared to IL-1β and IL-6, both of which, had little effect on CRIg expression^9^. IL-4 down regulated CRIg mRNA expression in MDM over a concentration range of 1–40 ng/ml, with a 60% reduction at 10 ng/ml (Fig. 6c). Decreased expression could be detected as low as 1–3 ng/ml concentrations of IL-4. IL-13 caused a reduction in expression of CRIg mRNA over a concentration range of 5–40 ng/ml (Fig. 6d).

The regulatory cytokine TGF-β1 caused a substantial decrease in CRIg mRNA over a concentration range of 2–15 ng/ml (Fig. 6e) and similarly for IL-10 over a concentration range of 5–40 ng/ml (Fig. 6f). In contrast treatment with dexamethasone increased CRIg expression (Fig. 6g). The colony stimulating factors differed in their effects on MDM CRIg expression. While GM-CSF down regulated expression, M-CSF caused an increase in expression (Fig. 6h and i).

### Effect of cytokines on CR3 and CR4 expression in macrophages

To gain a greater understanding of the consequences of cytokine-induced modulation of CRIg expression it is important to assess these changes relative to those induced in other functional receptors. Particularly important in this context is the expression of the classical complement receptors, CR3 and CR4, which also promote the phagocytosis of iC3b-opsonized particle^5, 10^. Thus the relative expression of these receptors may be a critical determinant of the severity of the inflammatory reaction. It was therefore considered important to understand whether CR3 and CR4 were also regulated by these cytokines and the type of changes the cytokines induced.

**Figure 7:**
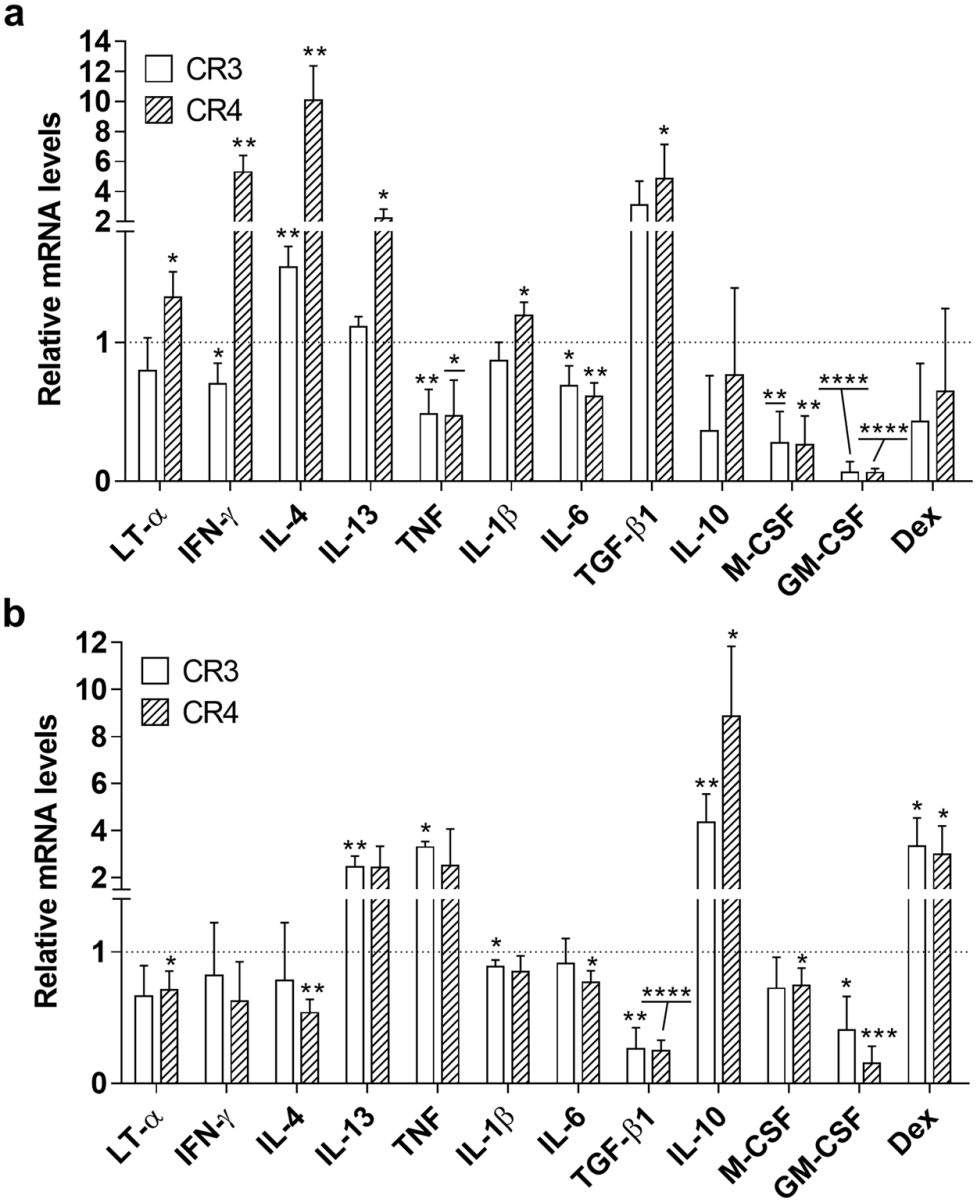
The effect of cytokines on the development of CR3^+^ and CR4^+^ macrophages and in MDM. (**a**) Monocytes were treated with 40 ng/ml LT-α, IFN-γ, IL-4, IL-13, IL-1β, IL-6, IL-10, M-CSF, GM-CSF or dexamethasone, 20 ng/ml TNF, 15 ng/ml TGF-β1. (**b**) Monocytes were cultured for 3 days for maturation into macrophages. The MDM were then incubated for 24 h with 40 ng/ml of the cytokines, LT-α, IFN-γ, IL-4, IL-1β, IL-6, IL-10, IL-13, M-CSF, GM-CSF, 20 ng/ml TNF, 15 ng/ml TGF-β1 or Dexamethasone (50 ng/ml). The level of CD11b and CD11c mRNA was measured using qPCR. Data are expressed as fold-change over GAPDH-normalized CD11b and CD11c mRNA in the absence of cytokine set as 1. Data are presented as means ± SD of three experiments, each conducted with cells from different individuals, *p < 0.05, *p < 0.05, **p < 0.01, ***p < 0.001, ****p < 0.0001.

The effect of cytokines on the development of CR3^+^/CR4^+^ macrophages from monocytes, as well as their direct effect on MDM was examined. In differentiating macrophages, the cytokines influenced the final expression of these complement receptors. In the majority of cases the increase or decrease in CD11b and CD11c mRNA caused by the cytokines were similar for CR3 and CR4 expression (Fig. 7a). The results demonstrated a clear decrease in CR3 and CR4 expression caused by TNF, IL-6, M-CSF and GM-CSF (Fig. 7a). In contrast several cytokines, while having no effect on CR3, increased expression of CR4 (Fig. 7a).

To examine the direct effects of cytokines on mature macrophages, the MDM were treated with cytokines and after 24 h the cells were examined for expression of CD11b and CD11c mRNA. The data showed that several of the cytokines had very little effect or decreased expression of these receptors (Fig. 7b). However IL-13, TNF and IL-10 caused an increase in CR3 and CR4 expression. Both M-CSF and GM-CSF reduced expression of these receptors (Fig. 7b). In contrast to the effects on developing macrophages (Fig. 7a), dexamethasone increased CR3 and CR4 expression in MDM (Fig. 7b).

### Effects of cytokines on macrophages phagocytosis of *C*. *albicans*

**Figure 8:**
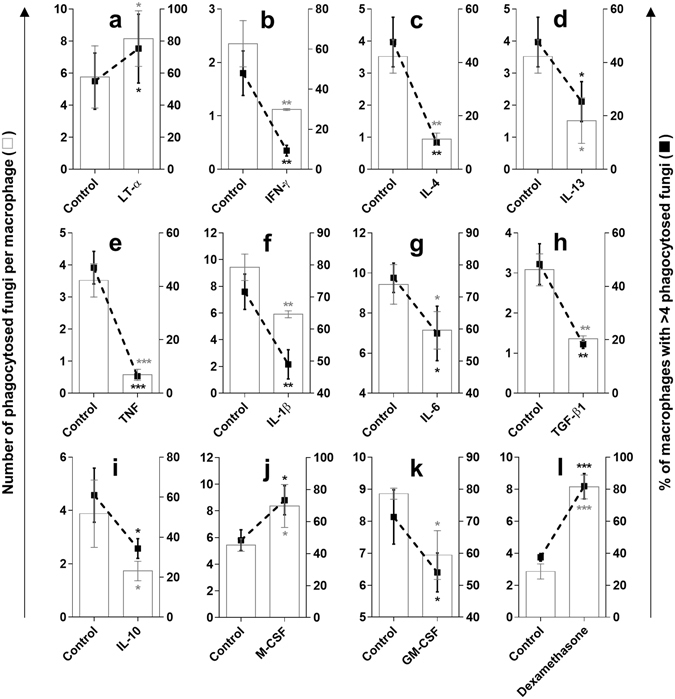
Effects of cytokines on the phagocytosis of *C*. *albicans* by MDM. MDM were prepared by culturing human monocytes for 5 days. The MDM were treated with 40 ng/ml of LT-α (**a**), IFN-γ (**b**), IL-4 (**c**), IL-13 (**d**), or 20 ng/ml TNF (**e**), or 40 ng/ml IL-1β (**f**), IL-6 (**g**), or 15 ng/ml TGF-β1 (**h**) or 40 ng/ml IL-10 (**i**), M-CSF/GM-CSF (**j**,**k**) or 50 ng/ml dexamethasone (**l**) for 24 h and examined for their ability to phagocytose complement opsonised *C*. *albicans*. Phagocytosis was scored as both the number of macrophages that had engulfed more than >4 fungi (line graph) and the number of fungi engulfed per cell (bar graph). Data are presented as means ± SD of three experiments, each conducted with cells from different individuals, *p < 0.05, **p < 0.01, ***p < 0.001.

To examine whether the effects of cytokines on CRIg expression in MDM corresponded to functional changes, we examined phagocytosis. In these experiments the MDM were treated with the cytokines for 24 h and were then challenged with *C*. *albicans* which had been opsonised with complement-containing human AB group serum. It has been established that *C*. *albicans* activates complement via the alternative pathway and that we see no phagocytosis when serum is heat inactivated^11, 12^. The data presented in Fig. 8 show that cytokine treatment of MDM altered their capacity to phagocytose *C*. *albicans*. While LT-α and M-CSF caused an increase in phagocytosis, all the other cytokines caused a decrease in fungal phagocytosis by the macrophages. This paralleled the effects of the cytokines on CRIg expression but not in the expression of CR3 and CR4. Similarly, dexamethasone which upregulated CRIg expression, increased the rate of phagocytosis of fungi by MDM (Fig. 8).

To gain further confidence in this correlation we examined CRIg protein expression by western blot analysis in the MDM which had been treated with cytokines for 24 h. The data presented in Supplementary Fig. S2 demonstrated that total CRIg protein expression, unlike the expression of CRIg mRNA, did not correlate with phagocytic activity of the cell, although the effects of some cytokines were consistent with mRNA levels. There was also no correlation with CD11b and CD11c protein expression. Because it has been previously reported that there are five different transcripts of CRIg, it is tempting to speculate that this may explain the discrepancy of the effects of cytokines seen at the mRNA and protein level. When we examined whether these transcripts were present in MDM, five were detected when the cells were stimulated with dexamethasone (Fig. S3). The antisera used only detected the L and S forms. As further antibodies to the different forms become available, this question will need to be revisited.

## Discussion

The data demonstrate that cytokines regulate the development of CRIg^+^ macrophages from monocytes, supporting and extending previous observations^8^ and the view that CRIg expression may be a control point in infection and immunity, through which cytokines control macrophage function. These cytokines could be divided into the group which promoted the development of CRIg^+^ macrophages, LT-α, IL-1β, IL-6, IL-10, GM-CSF, M-CSF and those which depressed this development, IFN-γ, TNF, TGF-β1, IL-4 and IL-13 (Table 1). This data not only identifies for the first time the cytokine patterns which regulate CRIg expression in macrophages but also reveal new and unexpected properties for some of these cytokines, which may have implications in the understanding of mechanisms of immunity to infection and in inflammation.

**Table 1:** Effect of cytokines on CR3/CD11b, CR4/CD11c and CRIg mRNA expression in macrophages.

Cytokine	During Macrophage Development			Expression in MDM		
	CR3/CD11b	CR4/CD11c	CRIg	CR3/CD11b	CR4/CD11c	CRIg
LT-α	↓	↑	↑	↓	↓	↑
IFN-γ	↓	↑	↓	↓	↓	↓
IL-4	↑	↑	↓	↓	↓	↓
IL-13	↓	↑	↓	↑	↑	↓
IL-10	↓	↓	↑	↑	↑	↓
TGF-β1	↑	↑	↓	↓	↓	↓
TNF	↓	↓	↓	↑	↑	↓
IL-1β	↓	↑	↑	↓	↓	↓
IL-6	↓	↓	↑	↓	↓	↓
M-CSF	↓	↓	↑	↓	↓	↑
GM-CSF	↓	↓	↑	↓	↓	↓
Dexamethasone	↓	↓	↑	↓	↓	↑

The ↑ and ↓ arrows represents an increase and a decrease in receptor expression.

Since a major and primary role of CRIg is to promote phagocytosis of bacteria^4–7^ our findings that cytokines can significantly alter the expression of CRIg suggest that the effects of these intercellular signalling molecules in infection and inflammation may occur via changes in CRIg expression. The data show that modulation of CRIg expression by cytokines is at a pre-transcriptional level and eventually emanates into corresponding changes in CRIg protein expression. Thus the effects of cytokines on CRIg protein expression by Western blot correlate with the changes seen at the mRNA level. The findings significantly extend the previous observation which only examined a restricted number of cytokines and which mainly assessed effects at CRIg mRNA level^8^.

While the effects of cytokines were found at concentrations that might be measured in septic patients they are untypically high for many inflammatory conditions such as rheumatoid arthritis (RA), general viral or bacterial infections). Potential technical reasons why higher than normal (*in vivo*) cytokine concentrations were required in these assays include protein absorption to the tubes. Although it is evident that in biological fluids even during inflammation that pg/ml and not ng/ml levels are found, in some fluids even levels up to 500ng/ml have been reported^13^. Other factors include, from our experience e.g. with TNF that detection in inflammatory fluids may not be indicative of the absolute cytokine levels as these are bound by tissue receptors especially as these seem to increase during an infection^14^. There may also be other serum/fluid factors which may cause measurement errors as well as cytokine decay, which we have experienced for biological fluids. It must also be appreciated that we are using recombinant cytokines, lacking glycosylation, and these may give different activities to the natural forms. Our use of concentrations between 2.5–40 ng/ml has followed other reports examining the effects of cytokines on macrophages. Perhaps one approach to resolving this issue is to look at levels produced by cells in culture following stimulation. Published data show that blood leukocytes stimulated with mitogens, bacteria and parasites produce ng/ml quantities of cytokines^15–17^. In conditions of severe pneumonia in patients serum levels of TNF and IL-6 reach ng quantities^18^.

The immuno-suppressive cytokine IL-10 caused a substantial increase in CRIg protein expression. In comparison another regulatory cytokine, TGF-β1, which shares properties with IL-10, profoundly decreased CRIg protein expression in developing macrophages. Our findings not only confirmed these results but also demonstrated a corresponding effect on CRIg protein expression. The two cytokines may thus form a regulation for CRIg expression in M2 macrophages in the killing of parasites^19–21^. While the pyrogenic cytokines, TNF, IL-1β and IL-6 share many biological activities, the effects on the development of CRIg^+^ macrophages differed. TNF caused a decrease and IL-1β and IL-6 increased CRIg expression. These changes were seen at both the mRNA and CRIg protein expression. Thus TNF versus IL-1β/IL-6 are likely to regulate CRIg expression in macrophages developing into M1 type^19–21^. Exposure to dexamethasone is likely to promote M2c macrophage development^20, 21^ with high CRIg expression (Table 1).

IFN-γ, IL-4, IL-10 and TGF-β1 altered CRIg expression, with both forms being affected. By measuring CRIg protein by Western blotting, the fate of both spliced forms of the receptor could be followed. The present studies revealed that the CRIg(L) and CRIg(S) were similarly, down- or up-, regulated by these cytokines. While both forms are found in human macrophages, murine macrophages possess only the latter^4^. Thus the finding that cytokines regulate the CRIg (S) form is also relevant to the murine models of infection and immunity and inflammation, since this is the form found in mouse macrophages.

Cytokine networks play an important role in regulating inflammation and those tested in our present study act on the macrophage, a cell which is central to infection and immunity, including immunity to *C*. *albicans* ^22^. Cytokines are known for their differences in either promoting disease or protecting against these diseases. It is tempting to speculate that CRIg may be one of the control points in infection and immunity through which cytokines and other intercellular acting inflammatory mediators act. Indirect support for this view can be derived from the findings that CRIg^+^ macrophages disappear from inflammatory sites and with the intensity of inflammation^23^.

It was interesting to find that both of the Th2 cytokines, IL-4 and IL-13 caused a decrease in expression of CRIg at the mRNA and protein level in maturing macrophages. This may be a mechanism by which macrophages promote pathogenesis induced by helminths such as schistosomes and other Th2 mediated inflammation such as that seen in allergy^21^. The observation could be given consideration in future research.

Often cytokines have been examined singly for their effects as this enables the contribution that the respective cytokine may have on cellular function. But to gain more physiological meaningful perspective we need to also understand the impact of cells interacting with the different cytokines simultaneously which may more closely mimic the *in vivo* inflammatory environment. To illustrate this we subjected monocytes during their development of CRIg^+^ macrophages to a combination of IL-1β, IL-6 and TNF. The depressive effects on CRIg^+^ macrophage development induced by TNF could be overcome by concomitant addition of IL-1β and IL-6. While the results suggest that during infection and inflammation the fluids generated are likely to increase the express ion of CRIg on macrophages, it is important to appreciate that the levels of these relative to each other will vary significant at different times of the inflammatory reaction, and may hence dictate the final outcome.

Here we have highlighted that cytokines not only affected the development of CRIg^+^ macrophages but also regulated the expression of this receptor on mature macrophages, indicative of events in tissues. However most of the cytokines caused a down-regulation of CRIg mRNA. Only LT-α and M-CSF induced an up regulation, similar to the anti-inflammatory agent dexamethasone. The findings indicate that mature macrophages are amenable to cytokine-induced modulation of CRIg (Table 1). This then becomes a second control point in inflammation through which cytokines may have their influence once the macrophages are matured and localized in tissues. The ability of LT-α and M-CSF to increase CRIg expression both during development and directly on mature macrophages is interesting. We have previously demonstrated that TNF caused these effects via activation of PKCα and those macrophages treated with anti-TNF antibody showed increased expression of CRIg^9^. It is therefore tempting to speculate that one important action of anti-TNF therapy is to prevent the loss of CRIg expression induced by TNF in RA and thereby improve phagocytic uptake of microbial pathogen, a possible reason as to why patients on anti-TNF therapy do not experience the expected wider increase in susceptibility to infection.

Cytokines which altered CRIg expression in macrophages, also caused changes to the expression of CR3 and CR4. It is evident from these results that some cytokines had opposite effects on these three receptor types (Table 1). The receptors, apart from performing similar functions, display other differing key functional properties. Thus their differential expression caused by cytokines will have an impact in the final response precipitated during microbial interaction. While IL-4 and TGF-β1 promoted the development of CR3 expressing macrophages, the development of CR4 expressing macrophages was promoted by the rest. Thus although CR3 may be decreased on macrophages subjected to LT-α, IFN-γ, IL-13 and IL-1β their phagocytic function is likely to be retained through the up regulation of CR4 by these cytokines. In comparison to this scenario, IL-4 and TGF-β1 promote the development of macrophages with increased expression of both CR3 and CR4; increasing the potential phagocytic capability of the macrophage. Although these are *in vitro* models, consideration should be given to these mimicking the monocyte invasion of tissue and their development into macrophages to interact with complement opsonised microbial pathogens, such as *Candida* ^24^. Macrophage development towards cells with lower phagocytic activity may occur when the same cytokines cause a decrease in expression of both CR3 and CR4. Cytokines which gave rise to this decrease were TNF, IL-10 and IL-6.

Although CR1 (CD35), is a complement control protein (CCP) module containing molecule, is present on the surface of macrophages, its role may not be to directly enhance phagocytosis of complement opsonized pathogens as opposed to the roles for CR3, CR4 and CRIg. In contrast, CR1 enhances clearance of soluble immune complexes via Fc receptors. CR1 on erythrocytes plays a major role in the clearance of soluble immune complexes, by transporting them to the liver and spleen, where they are cleared by macrophages. The binding of C3b-coated targets to phagocyte CR1 is not sufficient to trigger phagocytosis, but C3b–CR1 interaction enhances the FcγR-mediated phagocytosis of targets bearing both IgG and C3b^25–27^. When we examined the expression of CR1, it was evident that the cytokines did not alter the expression of this receptor (Fig. S2).

Examination of effects of cytokines on mature macrophages, MDM, demonstrated a different pattern of alteration in CR3 and CR4 mRNA (Table 1). The ability of cytokines to regulate these receptors provides a second check point for regulating macrophage function in infection and immunity, depending on the infection type and cytokines generated. The cytokines IFN-γ, TNF, IL-6, M-CSF and GM-CSF decreased the development of CR3^+^ macrophages. But this did not necessarily correspond to a similar effect on mature macrophages (Table 1). The findings show that CR3 and CR4 expression may be differentially regulated by some cytokines. Since Kupffer cells not only express CRIg but also CR3 and CR4^28^, the findings are also relevant to this tissue fixed macrophage. But further studies are required to ascertain whether this differential expression of CRIg versus CR3/CR4 induced by cytokines is also relevant to Kupffer cells.

Because most cytokines examined caused a decrease in CRIg expression on mature macrophages, it is inevitable that those monocytes which respond to infection in tissues and develop into macrophages will be susceptible to the action of these cytokines and this may be a reason why CRIg expressing macrophages are low at inflammatory sites and infection foci^23^. Previously we found that IFN-γ decreases the development of CRIg^+^ macrophages and caused reduced phagocytosis of complement opsonised *C*. *albicans* ^8^. The present study demonstrated that IL-4 caused a decrease in the expression of CRIg mRNA and reduction in the phagocytosis of *C*. *albicans*. We have previously reported that IL-4 caused a decrease in the phagocytosis and killing of complement-opsonised *Plasmodium falciparum* infected red blood cells by macrophages^29^. The changes in CRIg mRNA levels in MDM correlated with their altered rates of phagocytosis of complement opsonised *C*. *albicans*. Complement deposition on this fungi results from activation of complement via the alternative pathway^11, 12^. Complement opsonisation is required to see the effects of changes in CRIg expression^9^. Thus innate immunity may function through components of microbial pathogens stimulating human lymphocytes to produce LT-α^30^. As previously demonstrated by Helmy *et al*.^4^, once phagocytosis has been initiated by liver macrophages (Kupffer cells), CRIg expression is dramatically reduced. Our results indicate that this is most likely due to the release of cytokines, in particular TNF which decreases CRIg expression^9^.

Although we are emphasising a potentially important function for CRIg in the phagocytosis of fungi, the study has not been designed to conclusively prove this. Approaches such as blocking the receptor and or the other complement receptors would need to be under taken to establish their role in this function. Furthermore our results revealed that the MDM expression of CRIg protein neither correlated with expression of CRIg mRNA nor phagocytic activity. The most appropriate explanation for this discrepancy is that this anti-CRIg antibody only reveals the changes in the L and S forms. We identified five transcripts of CRIg in MDM and it is possible that changes in the expression of other forms may account for changes in rates of phagocytosis.

While our studies have focussed on phagocytosis of fungi, the importance of CRIg in phagocytosis of bacteria has been highlighted. Apart from implications in infections, our results suggest that cytokines may work through alterations in CRIg expression to modulate the inflammatory response in chronic inflammatory diseases such as RA. The pro-inflammatory, Th1 cytokine IFN-γ, in contrast to LT-α, causes a marked decrease in CRIg expression, in line with their reported effects in the pathogenesis of RA. IFN-γ is present in RA patients’ synovium and synovial fluid^31^. CD4 T cells in RA patients contribute to the pathogenesis by producing IFN-γ^32, 33^. Another Th1 cytokine, TNF, caused a decrease in CRIg expression. TNF is a major mediator of joint inflammation and bone destruction in inflammatory arthritis and several studies have measured large amounts of TNF in synovial fluid of patients with RA, psoriatic arthritis and in children with juvenile idiopathic arthritis^34–37^ TNF targeting biological drugs proved effective in the treatment of RA patients^38^. The role of LT-α, a close homolog of TNF^39^, found in synovial tissue of diseased joints, is not well defined^40^. In psoriatic arthritis patients, anti TNF-α monoclonal antibodies have been developed for neutralization of TNF and etanercept for LT-α^41^. Psoriatic arthritis patients undergoing etanercept treatment showed significantly increased serum levels of LT-α after 3 and 6 months which returned to baseline levels after 12 months^41^. These findings are conducive with our data showing that LT-α up regulated the expression of CRIg in macrophages. The difference between LT-α and TNF which act on the same receptor is not surprising as previously we have found that the two cytokines have some distinct biological effects on phagocytes^42^. For example in terms of mediating articular cartilage damage, LT-α plays a protective role compared to the destructive role of TNF^42^.

The immuno-suppressive cytokine IL-10 caused a substantial increase in CRIg mRNA and corresponding CRIg protein. This is consistent with its protective and anti-inflammatory effects observed in several murine arthritis models and its praised therapeutic potential in this disease^43, 44^. Another regulatory cytokine, TGF-β1 which shares properties with IL-10, however, plays a major role in the progression of RA and several studies reported that TGF- β1 has been detected in the synovial tissue of patients with RA^45, 46^. Our findings show that TGF-β1 which has regulatory effects on macrophages profoundly decreases CRIg mRNA^8^ and protein expression in macrophages and suggest that this may be a mechanism in the pathogenesis of RA. Although IL-1β is expressed in RA^47^, its role in inflammation has been controversial. Injection of recombinant IL-6 into the joint cavity reduced cartilage destruction in experimental arthritis^48^. Some studies reported that increases in serum IL-6 levels are associated with clinical improvements^49^. IL-6 reduces TNF production^50, 51^ which may explain its protective role in joint pathology. Our findings are in line with its protective effects by increasing CRIg expression.

It was interesting to find that the Th2 cytokines, IL-4 and IL-13 both caused a decrease in expression of CRIg at both the mRNA and protein level. It has been reported that there is an association of IL-4 gene 70 bp VNTR and MTHFRC677T polymorphism in the development of RA^52^. Furthermore, it has been suggested that IL-4 and its receptor could play a role in the pathogenesis of RA^53^. Similarly, IL-13 is also identified as a risk locus for psoriatic arthritis investigated in a number of studies^54^.

Because of the critical functions played by CRIg in infection and immunity and inflammation, our results suggest that cytokines have the potential to modify inflammation and resistance to microbial pathogens by modulating this receptor, hence identifying a mechanism by which cytokines regulate defence against infection and inflammation^55^. The research extended to show that cytokines could regulate the expression of CRIg on mature macrophages to provide a second control point by which cytokines could modify macrophage microbial killing, inflammation and immune responsiveness. Other classes of inflammatory mediators are likely to also regulate CRIg expression, as we previously found with arachidonate^8^. The importance of CRIg in Kupffer cell-mediated phagocytosis of bacteria has been demonstrated^4, 6, 7^ and it is likely that CRIg expression in these cells is also regulated by cytokines during infection and inflammation^56^. While the complexity of the CRIg system and its varied roles in infection and immunity is becoming appreciated^25^, we have now provided further evidence of its importance in host defence and understanding the mechanisms regulating macrophages in immunity to infection.

## Methods

### Cytokines and cell culture reagents

Recombinant granulocyte-macrophage colony stimulating factor (GM-CSF), macrophage (M)-CSF, interleukin (IL)-1β, IL-6, IL-4, IL-10, IL-13, interferon (IFN)-γ, lymphotoxin (LT)-α, tumor necrosis factor (TNF), M-CSF and GM-CSF were purchased from ProSpec-Tany Technogene (Rehovot, Israel), transforming growth factor (TGF)-β1 from R&D Systems (Minneapolis, MN), and dexamethasone was purchased from Sigma-Aldrich (St. Louis, MO). A mouse monoclonal antibody (clone 3C9) that recognizes the IgV domain of human CRIg was kindly provided by Dr. Menno van Lookeren Campagne (Genentech, San Francisco, CA). RPMI 1640 tissue culture medium, foetal calf serum (FCS) and L-glutamine were purchased from SAFC Biosciences (Lenexa, KS).

### Ethics statement

Venous blood was collected from healthy adult volunteers under guidelines and approval of the Women’s and Children’s Health Network Human Research Ethics Committee. Written informed consent was obtained from all participants.

### Purification and culture of monocytes

Peripheral blood mononuclear cells (PBMC) were prepared by centrifugation of blood on Ficoll-Paque Plus (GE Healthcare, Uppsala, Sweden). The interface layer containing PBMC was harvested and cells were washed in RPMI-1640 medium supplemented with 2 mmol/L L-glutamine, 100 U/ml penicillin, 100 µg/ml streptomycin and 10% foetal calf serum, pH 7.4 (RPMI-FCS). Cell viability was determined by the trypan blue-exclusion method. Monocytes were purified from the PBMC by density gradient centrifugation, as described previously^9^. Briefly, PBMC were layered onto a 46% iso-osmotic Percoll gradient (GE Healthcare, Uppsala, Sweden) and centrifuged at 600 × g for 30 min at room temperature. The monocytes-containing layer was harvested. Monocytes were >90% pure as judged by staining with anti-human CD14-FITC (BD Pharmingen, San Jose, CA) and analysing on a BD FACSCanto (BD Biosciences, San Diego, CA). Monocytes were cultured in RPMI-FCS in humidified air containing 5% CO_2_ at 37 °C at 10^6^ cells/ml under the influence of cytokines or dexamethasone. Cells were harvested after either 3 days (for CRIg mRNA analysis) or 7 days (for CRIg protein analysis) culture by gentle scrapping with a ‘rubber policeman’.

### Quantitative PCR

RNA was converted to cDNA using an iScript cDNA synthesis kit (Bio-Rad). QPCR was conducted using primers for human CD11b (Forward: CCTGGTGTTCTTGGTGCCC; Reverse: TCCTTGGTGTGGCACGTACTC), CD11c (F: CCGATTGTTCCATGCCTCAT; R: AACCCCAATTGCATAGCGG), and CRIg (F: ACACTTATGGCCGTCCCAT; R: TGTACCAGCCACTTCACCAA) with GAPDH (F: GAGTCAACGGATTTGGTCGT; R: GACAAGCTTCCCGTTCTCAGCCT) as the reference gene^8, 9^. Assayed in triplicate, each reaction contained 100 nM of each primer, 1 μl of cDNA, and iQ SYBR Green Supermix (Bio-Rad Laboratories) in a 20 μl final volume. Thermal cycling was performed with an initial denaturation at 95 °C for 5 min, followed by 40 cycles of 95 °C for 30 sec, 60 °C for 30 sec and 72 °C for 30 sec, using an iQ5 Real Time Detection System with iQ5 Optical System v2.1 software (Bio-Rad Laboratories). Expression data was normalised to GAPDH transcript levels.

### Western blotting

Macrophages were harvested after 7 days, washed, and resuspended in 100 μl of lysis buffer containing 20 mmol/L HEPES, pH 7.4, 0.5% Nonidet P-40 (v/v), 100 mmol/L NaCl, 1 mmol/L EDTA, 2 mmol/L Na_3_VO_4_, 2 mmol/L dithiothreitol, 1 mmol/L PMSF, and 10 μg/ml of each protease inhibitor (Benzamidine, leupeptin, pepstatin A and phenylmethylsulfonyl fluoride (PMSF) purchased from Sigma-Aldrich and aprotinin from Calbiochem (Merck, Darmstadt, Germany)^57^. Protein was quantitated by the Lowry method, prior to the addition of Laemmli buffer. Samples were boiled at 100 °C for 5 min and 60 μg of each were subjected to 12% SDS-PAGE at 175 V for approximately 1 h using the Mini-PROTEAN 3 system (Bio-Rad Laboratories, Hercules, CA). The samples were electrophoretically transferred to nitrocellulose membrane (Pierce Biotechnology, Thermo Fisher Scientific, Rockford, IL) at 100 V for 1 h. To monitor the extent of protein transfer, the membrane was stained with 0.1% Ponceau S (in 5% acetic acid). After blocking, the membrane was incubated with mouse anti-human CRIg (3C9) at 1:20000 in blocking solution overnight at 4 °C. Following washing in blocking solution (3 × 10 min), the membrane was incubated with secondary HRP-conjugated rabbit anti-mouse IgG (Dako, Glostrup, Denmark) at 1:2000 in blocking solution for 1 h at room temperature. Immunoreactive material was detected by enhanced chemiluminescence according to the manufacturer’s instructions (Western Lightning Chemiluminescence, Perkin Elmer, Waltham, MA). The protein bands on the membranes were visualised by a ChemiDoc XRS+ Imaging System and quantitated using Image LabTM Software, Version 3.0 (Bio-Rad Laboratories, Hercules, CA).

### Phagocytosis assay

The phagocytosis assay was performed essentially as described previously^8^. Twenty four hours post treatment of MDM with cytokine treatment, the cells were washed and detached with detachment buffer. Then 1 × 10^5^ *C*. *albicans* yeast particles were added to 5 × 10^4^ MDM in a final volume of 0.5 ml HBSS. Complement-containing human AB serum was added to a final concentration of 10%. The cells were incubated for 15 min at 37 °C on a rocking platform. Unphagocytosed yeast particles were removed by differential centrifugation at 175 × g for 5 min and then the MDM in the pellet were resuspended and cytocentrifuged onto a microscope slide and stained with Giemsa. The number of particles in phagocytic vacuoles was then determined^8^. Phagocytosis was scored as both the number of macrophages that had engulfed >4 fungi (line graph) as well as the number of fungi engulfed per cell (bar graph).

### Statistical analysis

Unpaired comparison were analysed using the two-tailed Student’s t-test and multiple comparison were performed using Dunnett’s test, with p < 0.05 considered significant.

## Electronic supplementary material

Supplementary Information
