# Supplementary material for: Cytokines regulate complement receptor immunoglobulin expression and phagocytosis of Candida albicans in human macrophages: A control point in anti-microbial immunity
Source: Sci Rep. 2017 Jun 22;7:4050. doi: 10.1038/s41598-017-04325-0 (PMC5481325; doi:10.1038/s41598-017-04325-0)
Supplement: Supplementary file 1 — Supplementary Information [file 41598_2017_4325_MOESM1_ESM.pdf]

Supplementary Information

Cytokines regulate complement receptor immunoglobulin expression and phagocytosis of *Candida albicans* in human macrophages: A control point in anti-microbial immunity

Usma Munawara[1,2,*]

Annabelle Small[2,*]

Alex Quach[2]

Nick N Gorgani[2,3]

Catherine A Abbott[1]

Antonio Ferrante[2,4]

[1]School of Biological Sciences, Flinders University; Bedford Park, South Australia; [2]Department of Immunopathology, SA Pathology at Women's and Children's Hospital; The Robinson Research Institute; Discipline of Microbiology and Immunology, University of Adelaide; [3]Children's Medical Research Institute, Westmead, New South Wales; [4]School of Pharmacy and Medical Sciences, University of South Australia, Adelaide, South Australia

*Equal contribution as first authors to the research.

Corresponding author: Professor Antonio Ferrante, Department of Immunopathology, SA Pathology, Women's and Children's Hospital, North Adelaide, South Australia, 5006. Email: antonio.ferrante@adelaide.edu.au

Supplementary Methods

Primers for CRIg transcript variants and CR1

Reverse-transcriptase PCR to detect five CRIg (*VSIG4*) transcript variants and SYBR Green qPCR for CR1 were performed using the primers and pairings listed in Table S1. The different CRIg variants are distinguished by their NCBI RefSeq Accession numbers. Each *VSIG4* primer was designed to anneal across either exons 3-4 or 7-8.

Supplementary Table S1. PCR primer sequences specific for CRIg transcript variants and CR1.

Gene/ Primer	Sequence (5' to 3')	Pairing	NCBI RefSeq	Transcript No.
VSIG4				
**F1**	TTTGTGGTCAAAGACTCCTCAAAGC	F1 and R1	NM_007268.2	1
**F2**	TGTCCAGAAACACTCCTCAAAGCT	F2 and R1	NM_001100431.1	2
**R1**	TGGCATGTGCCCTGGCT	F2 and R2	NM_001184831.1	3
**R2**	GAGAGACTTTCTTACCTGGCTGCTT	F1 and R2	NM_001184830.1	4
**R3**	GACACTTTGGGCTGGCTGCT	F1 and R3	NM_001257403.1	5
CR1				
**F**	CCCTTTGGAAAAGCAGTAAA			
**R**	TCAACTTGGCAAACAGAAAA			

VSIG4 primer F1 paired with R1 are specific for transcript variant 1, F2 with R1 for transcript variant 2, F2 with R2 for transcript variant 3, F1 with R2 for transcript variant 4, and F1 with R3 for transcript variant 5. All *VSIG4* primer pairings generate an amplicon of 292 bp in length. The primers for CR1 are from Anand et al (2014)[1] and expected to generate a 193 bp amplicon.

Supplementary Figures

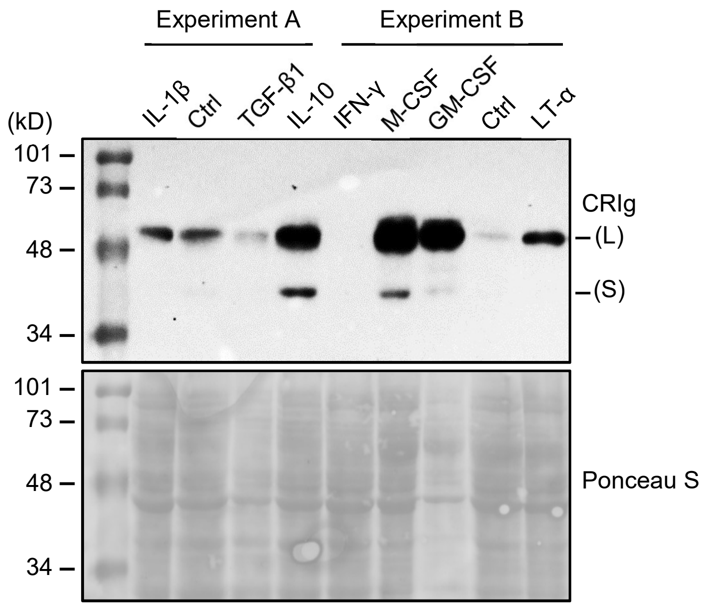

Supplementary Figure S1. Representative Western blot demonstrating molecular sizing of CRIg isoforms in cytokine-induced development of macrophages. The blot presents lysates from monocytes cultured in the presence of 40 ng/ml LT-α, IFN-γ, IL-1β, IL-10, M-CSF, GM-CSF, or 15 ng/ml TGF-β1 in separate individuals (experiment A and B) that were examined by staining with CRIg 3C9 monoclonal antibodies. The corresponding Ponceau S staining shows the consistency of protein load. Low Range Prestained SDS-PAGE Standards (Bio-Rad Laboratories) were used for determining the long (L) and short (S) forms of CRIg with ladder band sizes indicated in kilodaltons (kD).

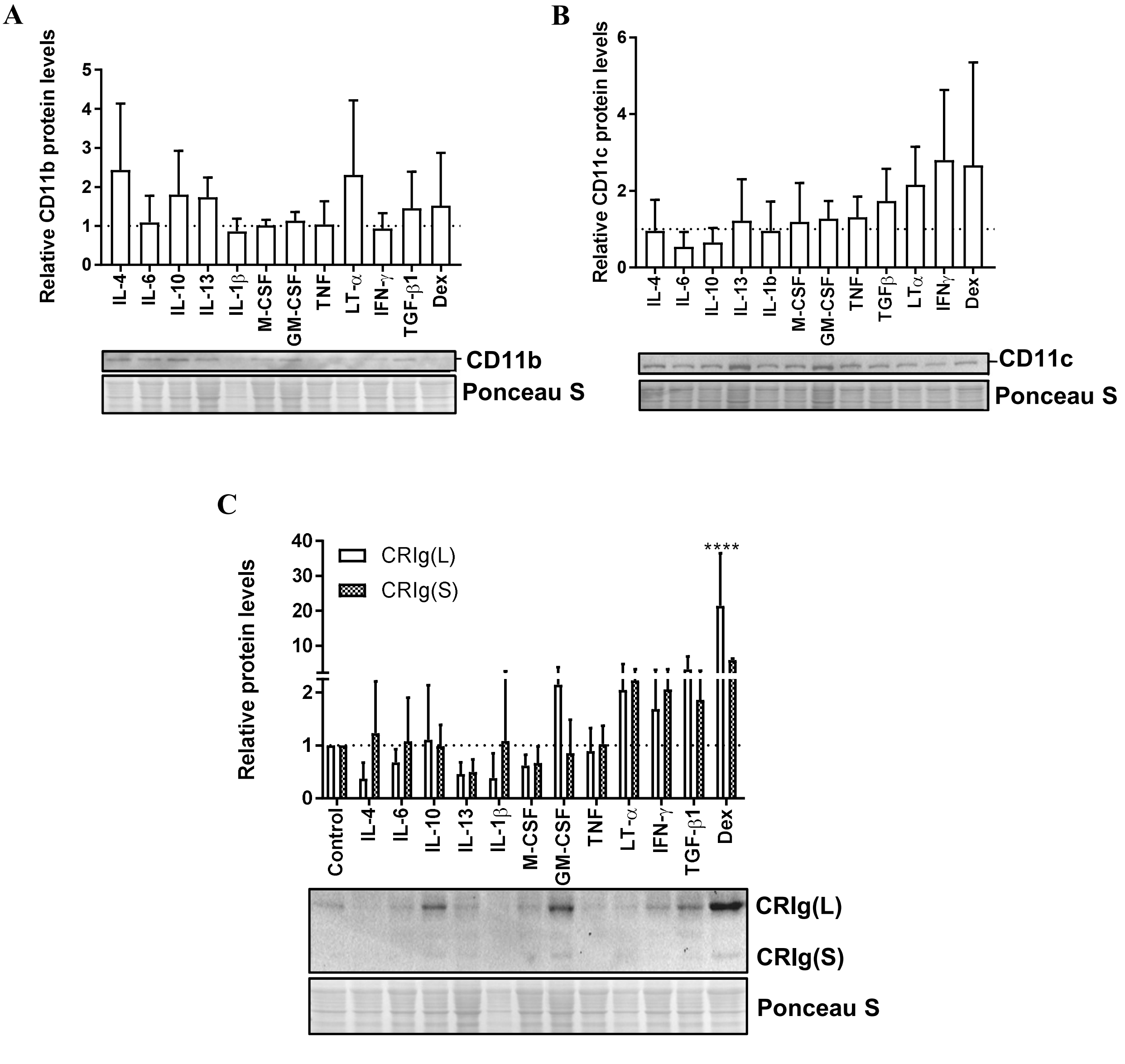

Supplementary Figure S2. Effects of cytokines on CRIg/CD11b/CD11c expression in matured macrophages (MDM). In these studies, MDM were prepared by culturing human monocytes for 7 days. MDM from 7 day cultures were treated with 40 ng/ml LT-α, IFN-γ, IL-4, IL-13, IL-1β, IL-6, IL-10, M-CSF, GM-CSF or dexamethasone, 20 ng/ml TNF, or 15 ng/ml TGF-β1 for 24 h and then (**a**) CD11b, (**b**) CD11c, (**c**) CRIg protein levels relative to Ponceau S loading control were assessed by Western blot. Data are normalised against untreated control cells and expressed as means ± SD of three experiments, each conducted with cells from different individuals.

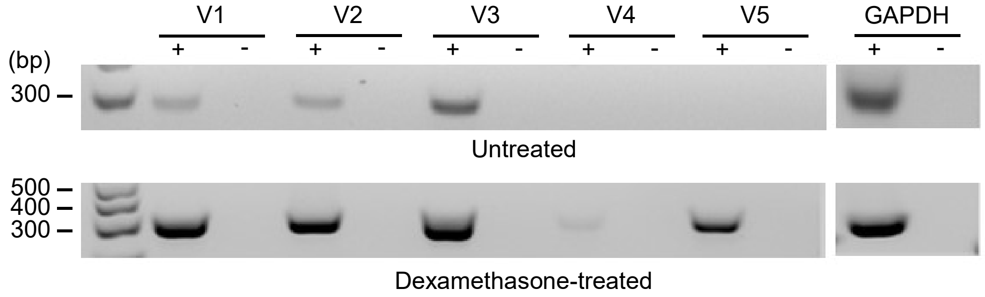

Supplementary Figure S3. CRIg transcript variants in macrophages. Agarose gel electrophoresis was used to visualise CRIg transcript variant amplicons generated from the cDNA of untreated macrophages (top row) and macrophages cultured for 3 days with 50 ng/mL dexamethasone (bottom row). The primers used are as shown in Table S1. Lanes labelled V1, V2, V3, V4 and V5 represent CRIg transcript variants 1, 2, 3, 4, and 5 respectively, with (+) indicating PCR with macrophage cDNA and (-) indicating PCR with no template. Amplification of GAPDH was used as an internal control. A 1kb Plus DNA Ladder (Invitrogen) was used to verify the size of the amplicons, with ladder band sizes indicated in base pairs (bp). Results are representative of three experiments.

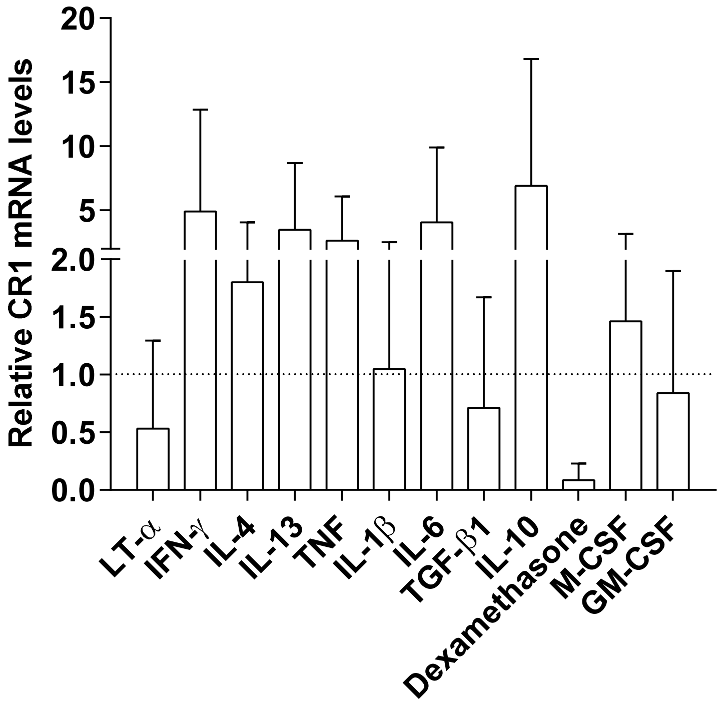

Supplementary Figure S4. Effects of cytokines on the development of CR1$^+$ macrophages. Monocytes were treated with 40 ng/ml LT-α, IFN-γ, IL-4, IL-13, IL-1β, IL-6, IL-10, M-CSF, GM-CSF or dexamethasone, 20 ng/ml TNF, 15 ng/ml TGF-β1, then CR1 mRNA expression measured. Data are normalised against untreated control cells and expressed as means ± SD of three experiments, each conducted with cells from different individuals.

Supplementary References

1 Anand, D., Kumar, U., Kanjilal, M., Kaur, S. & Das, N. Leucocyte complement receptor 1 (CR1/CD35) transcript and its correlation with the clinical disease activity in rheumatoid arthritis patients. *Clinical and experimental immunology* **176**, 327-335, doi:10.1111/cei.12274 (2014).
